# The association of ^18^F-FDG PET/CT and biomarkers in confirming coronary microvascular dysfunction

**DOI:** 10.1186/s13104-018-3892-6

**Published:** 2018-11-06

**Authors:** Henry Anselmo Mayala, Khamis Hassan Bakari, Abdalah Mkangala, Mafuru Magesa, Fabian Pius Mghanga, Wang ZhaoHui

**Affiliations:** 1 Department of Cardiology 10th Floor, Wuhan Union Hospital, Tongji Medical College of Huazhong University of Science and Technology, 501 Building, Hankou, Wuhan, 43000 Hubei Province China; 2 Department of Radiology and Nuclear Medicine, Wuhan Union Hospital, Tongji Medical College of Huazhong University of Science and Technology, Wuhan, Hubei China; 3 Department of Clinical Pharmacology, Wuhan Union Hospital, Tongji Medical College of Huazhong University of Science and Technology, Wuhan, Hubei China; 4 Department of Internal Medicine, Archbishop James University College, Songea, Tanzania

**Keywords:** Coronary microvascular dysfunction (CMVD), Brain natriuretic peptide (BNP), Red cell distribution (RDW), Coronary flow reserve (CFR)

## Abstract

**Objective:**

The purpose of this study is to evaluate the association between PET/CT CFR and biomarkers combined in confirming the diagnosis of coronary microvascular dysfunction.

**Results:**

A total of 28 patients (21 males and 7 females) were included in this descriptive observational study (both qualitative and quantitative). The mean patient age was 55.50 ± 10.21 years (range 27–70 years) and the median was 56.5 years (range 49–63 years). All patients underwent Echo, CAG and PET/CT scan. Chest tightness was the most common symptom in our study. Most patients had normal blood pressure (*n *= 18, 64.3%) while only (*n *= 10, 37.5%) had hypertension, and (*n *= 1, 3.6%) had diabetes mellitus. The mean HDL in CMVD (*n *= 25) and non-CMVD (*n *= 3) were 1.30 ± 0.39 and 1.08 ± 0.95, respectively, indicating that the difference between the groups was statistically significant (p = 0.04). Similarly, the mean HBA1c- (glycated haemoglobin) in CMVD (*n *= 25) and non-CMVD (*n *= 3) were 5.6 ± 0.53 and 5.0 ± 0.26, respectively, with (p = 0.03). Our findings managed to show the association between biomarkers and PET/CT CFR in confirming the diagnosis of coronary microvascular dysfunction.

## Introduction

Chest pain has been a common symptom in both cardiovascular and respiratory diseases or as referred pain in some of the gastrointestinal diseases. Chest pain as a cardiovascular symptom most commonly represents an important early indicator of either acute myocardial infarction or aortic dissection. Thus, early diagnosis and intervention has become particularly important in the management of cardiovascular diseases/emergencies.

Recent studies have shown that both obstructive epicardial coronary arteries and nonobstructive coronary microvascular disease have a similar fate of poor prognosis and a common entity of chest discomfort [1].

Coronary microvascular dysfunction is a clinical syndrome encompassing changes that lead to functional and structural abnormalities in the coronary microvasculature. These changes disrupt the ability of the vessels to vasodilate and augment coronary blood flow in response to increased myocardial demand, causing angina and ischemia [2].

Various studies have also revealed that 49% of patients who are undergoing clinically indicated coronary arteriogram do not have significant stenosis [3, 4]. Furthermore, 60% of these patients may have coronary microvascular dysfunction.

Previous study has indicated that, 59% of patients with anginal symptoms, but with angiographically normal coronary arteries, were found to have an abnormal response to vasodilator agents, adenosine, and acetylcholine, suggestive of coronary microvascular dysfunction [5]. Sade et al. [6], in their study which focused on angina-like chest pain and angiographically normal coronary arteries observed that 40% of study subjects had reduced CFR (mean, 1.7 ± 0.24) suggestive of microvascular dysfunction.

Hung et al. [7] in their study reported that, in healthy adults, an appropriate CFR cutoff value is over 3. Different biomarkers have been implicated in relation to the pathogenesis of coronary microvascular dysfunction namely CRP, LDL, HDL, BNP, troponin, red cell redistribution width etc. [7]. Inflammation and immune dysregulation play a pivotal role in endothelial dysfunction and CAD pathogenesis. Previous data have also documented the use of biomarkers in coronary microvascular disease [7]. The aim of this current study is to review and assess the value of F-FDG PET/CT combined with biomarkers in the diagnosis of CMVD.

## Main text

### Methodology

#### Study population

We assessed 28 patients admitted at department of cardiology, Wuhan union hospital, 26 who presented with typical angina symptoms and had a typical history of chest pain, ST-changes on EKG with normal coronary arteries on coronary angiography. 2 were control without any symptoms, no history of hypertension, diabetes mellitus or coronary artery disease, there ECG and CAG were normal.

#### Study type

A hospital based observational study.

### Study objective

The purpose of this study is to evaluate the association between PET/CT CFR and biomarkers combined in confirming the diagnosis of coronary microvascular dysfunction.

### Definition of terms

CMVD: Patients with CFR of < 2.6 which was considered abnormal.
Non-CMVD: Patients with CFR of ≥ 2.6 which was considered normal.

### Statistical analysis

Baseline patient characteristics were summarized. All data are presented as mean ± SD for continuous variables and n (%) for categorical variables. Comparisons between the groups were performed using student T-test for continuous variables and Chi square or Fisher exact test for categorical variables. Statistical analyses were done using IBM SPSS statistical software version 20.0. A p  < 0.05 was considered statistically significant.

### Positron emission tomography

#### Image acquisition

All patients fasted for at least 6 h before PET/CT examination. The images were obtained using a dedicated PET/CT scanner (Discovery VCT^®^, GE medical systems, Milwaukee WI, USA) 40–60 min after intravenous injection of 3.75–5.55 MBq/kg of 18F-FDG. A low dose CT scan was obtained for attenuation correction using: tube voltage 120 kV, 80 mAs, and 3.75 mm slice collimation. PET data were constructed with the ordered subset expectation maximization algorithm. Both CT and PET data were sent to a work station (Xeleris^®^, GE medical systems) for evaluation.

PET-CT scan was used to measure coronary flow reserve and assess the microvascular coronary perfusion.

## Results

A total of 28 patients (21 males and 7 females) were included in this descriptive observational study both qualitative and quantitative. The mean patient age was 55.50 ± 10.21 years (range 27–70 years) and the median was 56.5 years (range 49–63 years). A total of 16 patients had chest tightness, four chest pain and eight had mixed symptoms (chest tightness and chest pain). The characteristics of patients are summarized in Table 1.

**Table 1:** Showing patients demographic characteristics

Variables	Attribute	Frequency	Percentage
Gender	Male	21	75
	Female	7	25
Symptoms	Chest tightness	16	57.1
	Chest pain	4	14.3
	Mixed	8	28.6
Blood pressure	< 120/80 mmHg	14	50
	120/80–139/89 mmHg	8	28.6
	> 139/89	6	21.4
Smoking	Yes	6	21.4
	No	22	78.6
Alcohol	Yes	1	3.6
	No	27	96.4
Hypertension	Yes	10	35.7
	No	18	64.3
Diabetes mellitus	Yes	1	3.6
	No	27	96.4
New York Heart Association (NYHA)	I–II	24	85.7
	III–IV	4	14.3

Chest tightness was the most common symptom in our study. Most patients had normal blood pressure (*n *= 18, 64.3%) while only (*n *= 10, 37.5%) had hypertension, and (*n *= 1, 3.6%) had diabetes mellitus. Also, most of the patients in our study were in NYHA class I–II (*n *= 24, 85.7%).

The mean PET/CT CFR (PET/CT coronary flow reserve) was 2.0982 ± 0.55 (range 1.16–3.69). The mean LVEF (left ventricular ejection fraction) was 47.89 ± 12.57 (range 24–72%).

The mean HDL in CMVD (*n *= 25) and non-CMVD (*n *= 3) were 1.30 ± 0.39 and 1.08 ± 0.95, respectively, indicating that the difference between the groups was statistically significant (p = 0.04). The mean RDW (red cell distribution) in CMVD (*n *= 25) and non-CMVD (*n *= 3) were 13.9 ± 1.75 and 12.7 ± 0.32, respectively, indicating that the difference between the groups was statistically significant (p = 0.06). Similarly, the mean HBA1c- (glycated haemoglobin) in CMVD (*n *= 25) and non-CMVD (*n *= 3) were 5.6 ± 0.53 and 5.0 ± 0.26, respectively, suggesting that the difference between the groups was statistically significant (p = 0.03). Also, the mean BNP (Brain natriuretic peptide) in CMVD (*n *= 25) and non-CMVD (*n *= 3) were found to be 316.17 ± 526.35 and 42.17 ± 21.18, respectively, indicating that the difference between the groups was statistically significant (p = 0.02). The relationship of different biomarkers in relation to patients with CMVD and those without CMVD, the distribution of patients with CMVD and those without CMVD according CFR-PET values are presented in Table 2.

**Table 2:** Showing the mean and standard deviation of the biomarkers and imaging studies

Variable	N	Minimum	Maximum	Mean ± SD
Age	28	27	70	55.50 ± 10.21
LDL	28	0.94	3.48	2.25 ± 0.68
HDL	28	0.7	2.13	1.27 ± 0.37
TROPONIN	28	0.3	2030	164.90 ± 458.74
CRP	28	0	59.3	9.55 ± 13.31
RDW	28	12	18	13.75 ± 1.70
HBA1C	28	4.5	6.7	5.51 ± 0.54
BNP	28	0	2266	286.81 ± 503.73
ECHO	28	24	72	47.89 ± 12.57
PET-CFR	28	1.16	3.69	2.10 ± 0.55

The mean value of LDL in CMVD patients *n *= 25 (89.3%), and non-CMVD patients *n *= *25* (10.7%), were found to be similar, 2.21 ± 0.68 (95% CI − 1.24 to 0.47) and 2.21 ± 0.68 (95% CI − 1.24 to 0.47), respectively, hence statistical insignificant (p = 0.399). The mean values of different biomarkers of CMVD patients were found to be, low HDL 1.30 ± 0.39 (95% CI − 0.25 to 0.68) p = 0.04 [8, 9], high RDW 13.9 ± 1.75 (95% CI − 0.91 to 3.33), p = 0.06, high HbA1c 5.6 ± 0.53 (95% CI − 0.08 to 1.23) p = 0.03 and high BNP 316.17 ± 526.35 (95% CI − 316.2 to 909), p = 0.02, which were all statistical significant. For those who had CMVD, n = 15 had Type 1 CMVD and n = 10 had Type 2 CMVD.

## Discussion

The aim of this current study is to assess the association of F-FDG PET/CT combined with biomarkers in confirming the diagnosis of CMVD. Coronary flow reserve (CFR) is a non-invasive measure of coronary vasomotor function that integrates the hemodynamic effects of epicardial coronary stenosis, diffuse atherosclerosis and microvascular dysfunction on myocardial tissue perfusion [10]. CFR can be measured non-invasively by PET, Transthoracic Doppler echocardiography and cardiac MRI. We chose PET because dynamic PET imaging affords robust and reproducible measurements of absolute myocardial blood flow (MBF) in ml/min/g at rest and during pharmacological stress which allows the calculation of CFR (defined as a ratio between MBF at stress and MBF at rest [10, 11].

Our findings show that 57.1% of coronary microvascular dysfunction patients presented with chest tightness and not chest pain which is a typical presenting symptom of ischemic heart disease. We also found that all those patients who are smoking (24.1%) suffered from coronary microvascular dysfunction while 35.7% of the patients had hypertension. In addition, CMVD patients *n *= 25 (89.3%) presented with low HDL (cardio protective in CMVD patients) and high RDW, HBA1c, BNP while non CMVD patients *n *= 3 (10.7%) were found to have low LDL levels which were also like CMVD patients. Most of the patients in our study were male (75%), which is consistent with other previous literature of microvascular dysfunction [12]. Conflicting findings have also been reported regarding female sex and microvascular dysfunction. Recent studies reported an existence of association between female gender and increased microvascular dysfunction [13].

Kobayashi et al. [14] in their study observed that there was no sex difference in IMR (index of microcirculatory resistance) although CFR was lower in females than males possibly due to shorter resting thermodilution transit times in females, and female gender was an independent predictor in the decrease of CFR. Murthy et al. [15] in their study which used PET and CFR threshold < 2.0 found that microvascular dysfunction was common in both men and women (51% and 54%), respectively.

**Fig. 1:**
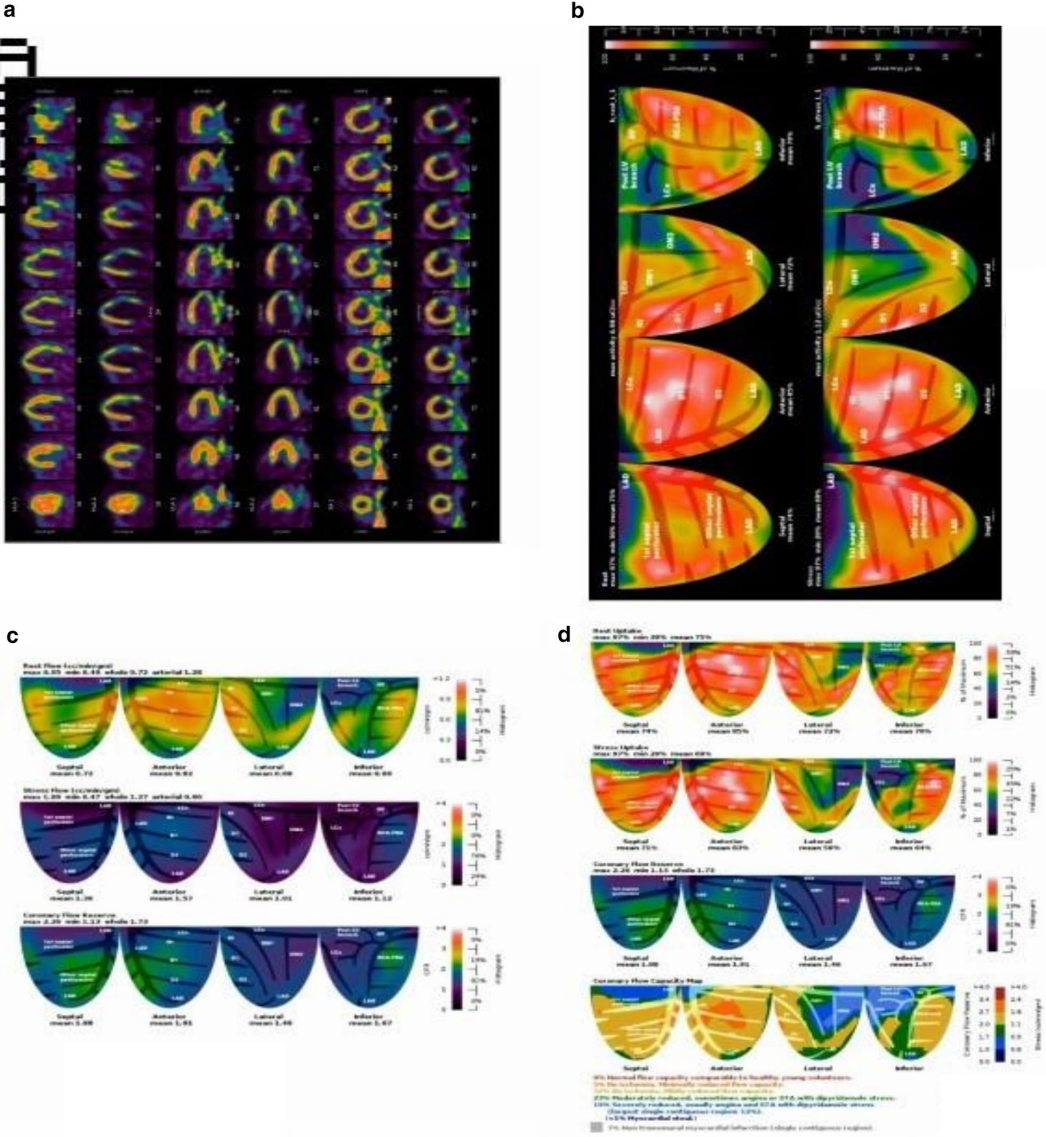
A 50 years old male patient with chief complain of chest tightness, his: Resting + ATP Load Pet myocardial perfusion imaging (**a**) and relative intake (**b**) showed left ventricular apical segment myocardial infarction, there were several segmental myocardial ischemias in different degrees between wall, lower wall and inferior wall, and absolute quantification (**c**) showed the absolute decrease of blood flow in the lower wall of left ventricle in resting state, the total and average blood flow of the left ventricle decreased, the left ventricle systolic function decreased, and the apical and lower wall movements were lower; comprehensive information is diagnosed as 1 type CMVD (coronary microvascular disease). **d** Showed regional and whole CFR

Most of our study subjects (89%) had reduced CFR < 2.6 with normal CAG findings thus confirming diagnosis of microvascular coronary artery disease and showing high sensitivity to PET imaging. Our findings above are congruent with previous published literatures which observed that patients with and without coronary artery disease (CAD) have CFR thresholds of 1.5–2.6 [16–22]. PET/CT images of 50 years old patient diagnosed as 1 type CMVD (Coronary microvascular dysfunction) as shown in Fig. 1.

Also, we found patients with abnormal CAG but with normal CFR indicating that they had no ischemic heart disease while we had patients with normal CAG but with abnormal CFR indicating they have ischemic cardiomyopathy. Hence, a misdiagnosis is likely which will eventually lead to mismanagement and finally resulting to poor prognosis because CMVD patients have almost equal complication like those with obstructive epicardial CAD. Therefore, it is important that we improve our knowledge on diagnosis of CMVD either by exploring more biomarkers especially cytokines and at the same time improve our knowledge on PET/CT CFR, to get an accurate diagnosis of CMVD and understand it more.

There are some limitations within our study. First, the low number of our study subjects and the cross-sectional nature of our study limit the magnitude at which our results can be used in a large clinical setting. Another limitation is the use of data from a single center. Also, symptom review was challenging since chest tightness is usually elucidated from the patients. Therefore, large prospective study with large sample size is required to validate our findings.

## Conclusion

PET/CT CFR examination combined with assessment of biomarkers such as, HDL, RDW, HBA1C, and BNP is very important in confirming the diagnosis of CMVD.

## Limitations

There are some limitations within our study. First, the low number of our study subjects and the cross-sectional nature of our study limit the magnitude at which our results can be used in a large clinical setting. Another limitation is the use of data from a single center. Also, symptom review was challenging since chest tightness is usually elucidated from the patients. Therefore, large prospective study with large sample size is required to validate our findings.

## Abbreviations

CFR: coronary flow reserve
PET: positron emission tomography
CAD: coronary artery disease
